# Decreased Levels of Histidine-Rich Glycoprotein in Advanced Lung Cancer: Association with Prothrombotic Alterations

**DOI:** 10.1155/2019/8170759

**Published:** 2019-03-03

**Authors:** Aleksandra Winiarska, Lech Zareba, Grzegorz Krolczyk, Grzegorz Czyzewicz, Michal Zabczyk, Anetta Undas

**Affiliations:** ^1^Institute of Cardiology, Jagiellonian University Medical College, Pradnicka 80, 31-202 Cracow, Poland; ^2^Faculty of Mathematics and Natural Sciences, University of Rzeszow, Rejtana 16C, 35-959 Rzeszow, Poland; ^3^Oncology Ward, John Paul II Hospital, Pradnicka 80, 31-202 Cracow, Poland; ^4^Faculty of Medicine and Health Sciences, Andrzej Frycz Modrzewski Krakow University, Herlinga-Grudzinskiego 1, 30-705 Cracow, Poland; ^5^Center for Research and Medical Technology, John Paul II Hospital, Pradnicka 80, 31-202 Cracow, Poland; ^6^Faculty of Medicine and Health Sciences, Jan Kochanowski University, IX Wiekow Kielc 19A, 25-317 Kielce, Poland

## Abstract

**Background:**

Histidine-rich glycoprotein (HRG) displays anticoagulant and antifibrinolytic properties in animal models, but its effects in humans are unclear. We investigated serum HRG levels and their associations with the disease stage and prothrombotic alterations in lung cancer (LC) patients.

**Methods:**

In 148 patients with advanced LC prior to anticancer therapy (87 non-small-cell LC and 61 small-cell LC) versus 100 well-matched controls, we measured HRG levels in association with clot permeability (*K*_s_), clot turbidimetry (lag phase and maximum absorbance), and clot lysis time (CLT).

**Results:**

Compared to controls, LC patients had 45.9% lower HRG levels with no associations with demographics and comorbidities. Decreased HRG, defined as the 90^th^ percentile of control values (<52.7 *μ*g/ml), was 16 times more common in subjects with than without LC (OR = 16.4, 95% CI 9.2-23.5, *p* < 0.01). HRG < 38 *μ*g/ml discriminated stage IIIAB/limited disease from IV/extensive disease (ED) LC. In LC patients, HRG correlated inversely with CLT (*r* = −0.41, *p* < 0.001), but not with other fibrin variables. Among stage IV/ED LC, HRG correlated significantly with *K*_s_ and lag phase (*r* = 0.28 and *r* = 0.33, respectively, both *p* < 0.001). LC patients with low *K*_s_ (10^th^ percentile of control values) combined with prolonged CLT (90^th^ percentile of control values) had reduced HRG levels compared to the remainder (*p* = 0.003). No such observations were noted in controls.

**Conclusions:**

Our study is the first to show that decreased HRG levels occur in advanced LC and are associated with the disease stage and hypofibrinolysis.

## 1. Introduction

Lung cancer incidence has raised from an infrequent entity in the year 1912 to one of the most commonly occurring cancers over the past century [1]. In 2012, almost 1.82 million new cases have been diagnosed, representing approximately 12.9% of all cancers worldwide [2]. It is estimated to account for almost every fifth death (1.59 million deaths per year, 19.4% of the total) [2]. Despite the introduction of new therapies such as chemotherapy and immunotherapy that prolong the lives of patients and in some cases make cancer a chronic disease, lung cancer remains the most prevalent fatal tumor [2, 3].

Venous thromboembolic disease (VTE) is common in subjects with active cancer with the prevalence of 20% in this disease [4]. Approximately 3% of lung cancer patients experience a VTE episode within two years after diagnosis, which is associated with a higher mortality for both non-small-cell lung carcinoma (NSCLC) and small-cell lung carcinoma (SCLC) [5]. Prothrombotic mechanisms resulting in VTE occurrence among lung cancer patients include enhanced expression and release of tissue factor, higher levels of thrombin-antithrombin complexes and plasmin-alpha 1-antiplasmin complexes, increase in platelet count, and elevated levels of procoagulant factors such as lupus anticoagulant, anticardiolipin antibodies to factor VIII, interleukin-6, and tumor necrosis factors [6].

Plasma fibrin clots are formed as ultimate products of thrombin-mediated conversion of fibrinogen into fibrin. Genetic and environmental factors affect the formation of compact, highly branched networks with thin fibers resistant to fibrinolysis [7, 8]. Fibrin clot properties are unfavorably altered by cigarette smoking [9], a well-established risk factor for nearly 90% cases of lung cancer [10]. Among patients with malignancy, subjects with digestive tract cancer [11] and multiple myeloma [12] were characterized by a prothrombotic clot phenotype. There is evidence for pathological clot microstructure with changes of physical properties, including higher fractal dimension in patients with extensive than those with localized lung cancer [13].

Since its discovery in 1972 [14], histidine-rich glycoprotein (HRG), an abundant plasma protein produced in the liver and circulating at a concentration of 100–150 *μ*g/ml [15], remains poorly characterized in terms of its role in human pathophysiology. Plasma HRG concentrations increase with age but decrease in acute inflammation, chronic autoimmune diseases, advanced liver cirrhosis, after renal transplantation, in asthma and chronic obstructive pulmonary disease treated with corticosteroids, and in acquired immune deficiency syndrome [15, 16]. Reduced HRG levels have been found in patients with ovarian cancer and hepatocellular carcinoma [17, 18]. HRG binds to heparin, which prevents the formation of heparin-antithrombin complexes and results in inhibition of the antithrombin activity [19]. It is estimated that nearly 50% of plasminogen circulates bound to HRG, which reduces plasminogen available to bind fibrin [19, 20]. Moreover, HRG can be incorporated to fibrin clots and its presence causes the formation of thinner fibrils as demonstrated in a purified system [20]. HRG-deficient mice, compared to control, showed higher spontaneous fibrinolytic activity but shorter prothrombin and bleeding time, indicating both prothrombotic and antifibrinolytic properties [21]. A role of HRG in human hemostasis is unclear. There is evidence for an active involvement of HRG in cancer largely by displaying both pro- and antiangiogenic properties [22–25]. Moreover, HRG by promoting tumor-associated macrophage polarization into an antitumor phenotype [24, 25] may result in tumor growth inhibition and it is a possible mechanism of cancer progression. Available data on HRG in cancer patients is inconsistent [26–28]. To our knowledge, no reports on HRG in lung cancer and its role in prothrombotic alterations have been published until now. Based on the available data, we hypothesized that HRG levels are reduced in advanced lung cancer in association with the disease severity and they contribute to unfavorable fibrin clot properties.

## 2. Materials and Methods

### 2.1. Patients

From May to September 2014, 148 consecutive adult patients with advanced lung cancer prior to anticancer treatment were recruited at the John Paul II Hospital in Cracow, Poland. This cohort was part of the study population reported previously in detail [29]. Briefly, lung cancer was confirmed and classified with the applicable histological classification of lung cancer according to the World Health Organization with the International Association for the Study of Lung Cancer modification for adenocarcinoma. Cancer stage was determined according to the American Joint Committee on Cancer 7^th^ edition staging scheme. Controls (*n* = 100) matched for age, sex, and cardiovascular risk were recruited using the same screening protocol and included members of the hospital personnel and their relatives. The exclusion criteria for both groups were as follows: any infections, glomerular filtration rate < 60 ml/min, hypo- or hyperthyroidism, any acute vascular events, acute venous thromboembolism, and current anticoagulant therapy except for prophylactic doses of low-molecular-weight heparin administered for the last time 12 h or more prior to the sample collection.

The research has been complied with all the relevant national regulations and institutional policies and in accordance with the tenets of the Helsinki Declaration and has been approved by the local ethical committee. A written informed consent was obtained from each participant.

### 2.2. Laboratory Investigations

Fasting blood samples were drawn from an antecubital vein with minimal stasis between 8:00 and 11:00 AM. Routine laboratory techniques were applied to determine blood cell count, creatinine, and D-dimer. Fibrinogen was determined using the Clauss method. Plasminogen was measured by a chromogenic assay (STA Stachrom; Diagnostica Stago, Asnieres, France). HRG was measured using an ELISA according to the manufacturer's instruction (Cusabio Technologies, US).

### 2.3. Plasma Fibrin Clot Analysis

In citrated plasma (vol/vol, 9 : 1 of 3.2% sodium citrate), the following variables describing a plasma clot formation, structure, and lysability were determined in duplicate by technicians blinded to the origin of the samples (intra-assay and interassay coefficients of variation, 5% to 7%). Details of the methodology used were presented previously [30].

#### 2.3.1. Clot Permeability

Permeation of plasma fibrin clots was determined as described [31]. Briefly, 20 mM calcium chloride and 1 U/ml human thrombin (Sigma-Aldrich, St. Louis, MO, USA) were added to citrated plasma. Tubes containing the clots were connected to a reservoir of a Tris-buffered saline (TBS; 0.01 M Tris, 0.1 M NaCl, pH 7.4), and its volume flowing through the gels was measured. A permeation coefficient (*K*_s_), which indicates the pore size, was calculated from the following equation: *K*_s_ = *Q* × *L* × *η*/*t* × *A* × Δ*p*, where *Q* is the flow rate in time *t*, L is the length of a fibrin gel, *η* is the viscosity of liquid (in poise), *t* is percolating time, *A* is the cross-sectional area (in cm^2^), and Δ*p* is a differential pressure (in dyne/cm^2^).

#### 2.3.2. Turbidity Measurements

Plasma citrated samples were mixed at a ratio of 2 : 1 with a TBS containing 0.6 U/ml human thrombin (Sigma-Aldrich) and 50 mM CaCl_2_ to initiate polymerization. Absorbance was read at 405 nm with a Perkin-Elmer Lambda 4B spectrophotometer (Molecular Devices). The lag phase of the turbidity curve, which reflects the time required for initial protofibril formation, and maximum absorbance at the plateau phase (ΔAbs), indicating the number of protofibrils per fiber, were recorded [31].

#### 2.3.3. Clot Lysis Assay

Clot lysis time (CLT) was measured as described previously [31]. Briefly, to 75 *μ*l of citrated plasma we added TF (dilution 105 times; Innovin, Dade Behring, Deerfield, IL, USA), CaCl_2_ (final concentration, 17 mM), tissue plasminogen activator (tPA, final concentration, 30 U/ml; Boehringer Ingelheim, Ingelheim, Germany), and phospholipid vesicles [32] (final concentration, 10 mM). HEPES buffer was added to make a total volume of 150 *μ*l.

### 2.4. Statistical Analysis

Data management was performed with STATISTICA 10.0 software (StatSoft Inc. 2011, Tulsa, OK, USA). Normal distribution for continuous variables was checked using the Shapiro-Wilk statistics. Continuous variables were presented as mean ± SD for normally distributed variables and as a median and interquartile range for nonnormally distributed variables. Proportional variables were compared by *χ*^2^ test and Student's *t*-test while continuous variables by Mann-Whitney *U*-test (Kruskal-Wallis and multiple repetition tests), as appropriate. Pearson's rank correlation coefficient was used to evaluate an association between two variables. To test the relationship between continuous variables, a univariate linear regression model was used. Because HRG had a skewed normal distribution, a 10 log-transformed data was entered for a better fit the model. Other variables were calculated on the original scale. Independent determinants of HRG were established in a multiple regression model, built by a forward stepwise selection procedure, verified by Snedecore's *F* statistics, with *F* > 1. The *R*^2^ was used as a measure of the variance. Log-linear analysis and unconditional multivariate logistic regression were performed to make adjustments for categorical variables (age, body mass index, and gender) [30]. To calculate odds ratios (ORs) with 95% CIs, *K*_s_, CLT, and HRG values were divided using the value of 90^th^ percentile in controls as a cut-off point. The best cut-off value that maximizes sensitivity and specificity and differentiates lung cancer patients from control subjects was calculated by using the receiver operating characteristic (ROC) curve. The significance level was set at *p* < 0.05.

## 3. Results

### 3.1. Patient Characteristics

A final analysis included 148 lung cancer patients and 100 matched control subjects (Table 1). There were 58 (39.19%) subjects diagnosed with SCLC, including 33 patients with limited disease and 25 patients with extensive disease, and 90 (60.81%) subjects with NSCLC (among them 24 with IIIAB stage/locally advanced inoperative neoplasm and 66 with IV stage/metastatic disease). As expected the lung cancer patients had lower BMI and were more often smokers (79 vs. 10; Table 1). They also had higher white blood cells (WBC), lower hemoglobin, and higher platelet count (Table 1). The cancer group had elevated D-dimer and fibrinogen. Among fibrin clot variables, lowered *K*_s_ (-26.52%), prolonged lag phase (+8.24%), increased ΔAbs (+6.25%), and longer CLT (+31.13%) were detected in lung cancer patients. The study groups did not differ in plasminogen levels.

### 3.2. HRG Serum Level Associations

**Figure 1:**
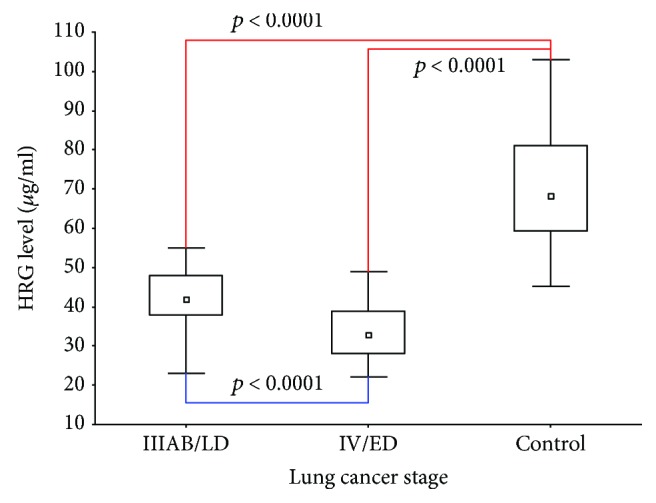
Distribution of histidine-rich glycoprotein (HRG) serum levels in controls and lung cancer patients with stage IV/extensive disease and locally advanced inoperative stage IIIAB/limited disease. Data are presented as median, interquartile range, and maximum and minimum values. Numbers on the graph represent *p* values in comparison to control and between lung cancer patient groups. LD: limited disease; ED: extensive disease.

Lung cancer patients had 45.87% lower HRG level compared to control subjects (Figure 1). Serum HRG showed no associations with demographic variables. Comparing histological subtypes of LC in the studied cohort, we observed that patients diagnosed with adenocarcinoma (*n* = 41) had lowered HRG (median, 32 (28-40) *μ*g/ml vs. 39 (32-44) *μ*g/ml, *p* = 0.002). Reduced HRG levels were detected in patients with metastases (*n* = 101, 33.8 ± 7.6 vs. 41.4 ± 7.8 *μ*g/ml, *p* = 0.044). Metastatic cancer was also associated with prolonged CLT (>105 min, OR = 2.09, 95% CI (1.18-3.73), *p* = 0.01), but not with other fibrin variables.

In the lung cancer group, HRG was inversely correlated with D-dimer (*r* = −0.37, *p* < 0.001) and fibrinogen (*r* = −0.18, *p* < 0.001), while it was positively correlated with lag phase (*r* = 0.20, *p* < 0.001). *K*_s_ and ΔAbs showed no associations with HRG in cancer patients, however CLT was inversely correlated with HRG (*r* = −0.38, *p* < 0.001). In the stage IIIAB/LD lung cancer patients, lowered HRG was associated with longer CLT (*r* = −0.41, *p* < 0.001), while in more advanced stage IV/ED, reduced HRG was associated with higher D-dimer (*r* = −0.35, *p* < 0.001), shorter lag phase (*r* = 0.33, *p* < 0.001), decreased *K*_s_ (*r* = 0.28, *p* < 0.001), and prolonged CLT (*r* = −0.5, *p* < 0.001). No such correlations were detected for HRG and fibrinogen or plasminogen.

**Figure 2:**
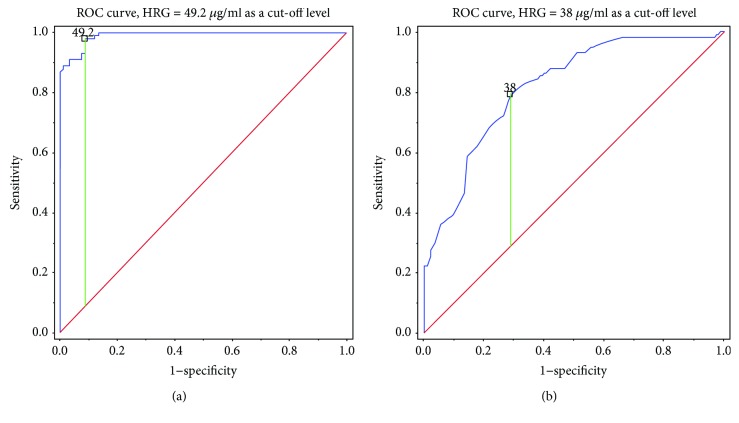
Receiver operating characteristic (ROC) curve for serum levels of histidine-rich glycoprotein (HRG) to discriminate between lung cancer patients and healthy subjects (a) and between IIIAB/limited disease and IV/extensive disease lung cancer (b). The blue line represents serum HRG level and the red line represents the reference line. The best cut-off point of HRG at 49.2 *μ*g/ml (a) and 38 *μ*g/ml (b) is marked, *P* < 0.0001.

Low HRG levels defined using the 90^th^ percentile of control values (i.e., 57.2 *μ*g/ml) observed in 143 (94.08%) lung cancer patients were detected in all patients diagnosed with stage IV/ED lung cancer. Metastatic stage IV/ED lung cancer patients had significantly decreased HRG compared to locally advanced lung cancer (stage IIIAB/LD) subjects (*p* < 0.0001, Figure 1). HRG cut-off levels which discriminate lung cancer patients from healthy subjects and stage IIIAB/LD from stage IV/ED lung cancer were below 49.2 *μ*g/ml (sensitivity, 98% and specificity, 90%) and below 38 *μ*g/ml (sensitivity, 80% and specificity, 70%) (Figure 2), respectively. HRG below 38 *μ*g/ml was associated with stage IV/ED lung cancer (OR = 3.07, 95% CI 2.08-4.54, *p* < 0.01).

**Figure 3:**
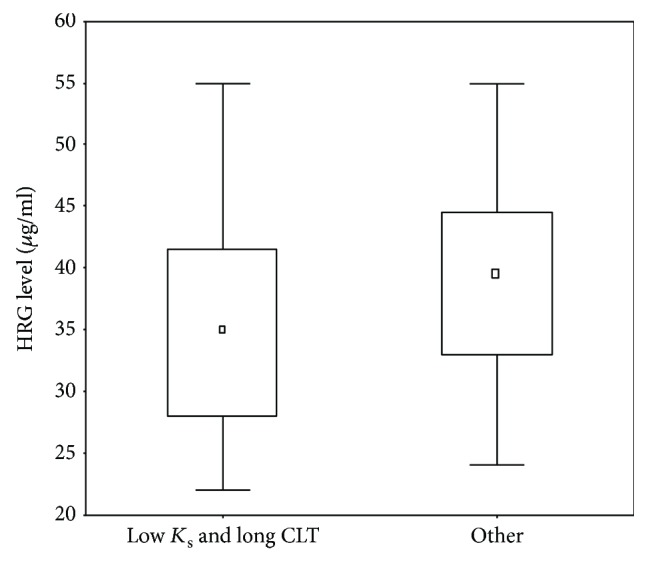
Distribution of histidine-rich glycoprotein (HRG) levels among lung cancer patients who formed compact fibrin clots (defined as the low value of fibrin clot permeability coefficient, 1^st^ quartile in the control subjects) and clots resistant to lysis (defined as long clot lysis time, 4^th^ quartile in the control group) compared to other subjects. Data are presented as median, interquartile range, and maximum/minimum values. *p* = 0.003. *K*_s_: fibrin clot permeability coefficient; CLT: clot lysis time.

A multiple logistic regression analysis showed associations between HRG levels and *K*_s_ as well as CLT in lung cancer patients (Table 2), also after adjustment for aspirin use (data not shown). In the combined analysis, compact fibrin clots (1^st^ quartile of control values of *K*_s_) and hypofibrinolysis (4^th^ quartile of control values of CLT) were more often found in subjects with lung cancer (OR = 3.08, 95% CI 2.29-4.14 and OR = 2.95, 95% CI 2.2-3.95, respectively). Among patients who had HRG below 52.7 *μ*g/ml, 62.24% of them formed dense and poorly lysable fibrin clots (Figure 3).

## 4. Discussion

The present study is the first to show associations between unfavorably altered fibrin clot properties and serum HRG level in advanced lung cancer patients. Moreover, we observed lower HRG levels in this patient group in association with more advanced cancer stage. Lower HRG was linked to reduced clot permeability and impaired susceptibility to lysis, which might contribute to thromboembolic events in cancer patients [33]. The study increases the current knowledge on the role of HRG in lung cancer by showing that the lower the HRG, the more advanced the disease stage and the more altered the clot features. Existing evidence indicates that HRG represents an adaptor protein with the potential to modulate immune, vascular, and coagulation systems, which are involved in cancer development. In the tumor environment, HRG promotes tumor-associated macrophage polarization into an antitumor (M1) phenotype [24, 25]. The presence of HRG normalizes tumor vasculature, allowing cytotoxic T-lymphocytes, natural killers, and dendritic cells to infiltrate into its microenvironment [24, 25], resulting in tumor growth inhibition. HRG released from platelets have been shown to promote angiogenesis in cancer by interfering with thrombospondin 1 and thrombospondin receptor CD36-mediated antiangiogenic signaling [22, 23]. Other studies have postulated an antiangiogenic activity of HRG. In mice, HRG deficiency was associated with reduced expression of antiangiogenic chemokines such as chemokine C-X-C motif ligand 10 and chemokine C-X-C motif ligand 11 and also decreased vessel coverage and perfusions [24]. In HRG-treated mice, tumor vascularization was inhibited [25]. Proteomics study using isobaric tagging and LC-MS/MS analysis revealed a significant reduction in serum HRG associated with progression of hepatitis B virus-related hepatocellular carcinoma [18]. Wang et al. observed downregulation of serum HRG precursor by LC-MS/MS in patients with atypical endometrial hyperplasia compared to normal donor controls, indicating that lower HRG levels lead to endometrial cancer progression [27]. Wu et al. [17] assessed ovarian cancer patients and showed decreased HRG levels in patients with stage I/II as well as with stage III ovarian cancer compared to healthy controls [17]. HRG levels may also be increased in some malignancies as reported by Matboli et al. who observed higher HRG levels in breast cancer (median, 17.1 mg/dl) compared to benign breast lesions (median, 1.56 mg/dl) and the control (median, 1.1 mg/dl) group [26]. Ahmed et al. [28] have observed in acute lymphoblastic leukemia that both HRG serum levels and protein RNA expression decreased during the leukemia treatment. Taken together, the present study shows that advanced lung cancer is associated with decreased HRG levels, which appears to be a common feature in most advanced cancers. A novel observation is that prothrombotic alterations are related to this decrease.

This study has several limitations. First, the sample size was limited and HRG levels were measured at a single time point. Second, we did not measure several potential fibrin clot modifiers including homocysteine, which were beyond the scope of the study. Third, the correlations demonstrated in the present study may not necessarily mean cause-effect relationships. Fourth, the current data cannot be necessarily extrapolated to patients with stages I and II lung cancer. Finally, *in vitro* experiments to better characterize the function of HRG in lung cancer patients, including HRG binding to heparin and fibrin clots [34–36], should be performed in the future. It is also worth investigating whether HRG levels and fibrin properties in lung cancer patients are associated with inflammation and cellular senescence [37].

## 5. Conclusions

The current study shows that decreased HRG levels occur in advanced lung cancer and are associated with the disease stage and prothrombotic plasma clot characteristics, including faster formation of more compact clots and impaired susceptibility to lysis. Clinical relevance of these findings needs further investigation.

## Figures and Tables

**Table 1:** Demographic, clinical, and laboratory characteristics of lung cancer patients and control subjects.

Variable	Lung cancer patients (*n* = 148)	Control subjects (*n* = 100)	*p* value
Age (years)	65 (46-82)	63 (43-81)	0.49
Male, *n* (%)	30 (20.3)	20 (20.0)	0.08
Body mass index (kg/m^2^)	25.0 (14.6-40.6)	26.9 (19.9-36.2)	<0.0001
*Clinical features*			
Current smoking, *n* (%)	53 (35.8)	10 (10.0)	<0.0001
Arterial hypertension, *n* (%)	48 (32.4)	55 (55.0)	0.3
Diabetes mellitus, *n* (%)	12 (8.1)	7 (7.0)	0.19
Previous stroke, *n* (%)	4 (2.7)	8 (8.0)	0.09
*Medications*			
Statin, *n* (%)	26 (17.6)	42 (42.0)	0.004
Aspirin, *n* (%)	25 (16.9)	38 (38.0)	0.005
*Laboratory investigations*			
WBC (10^3^/*μ*l)	9.2 (8.4-10.0)	6.0 (5.8-6.2)	<0.0001
Hemoglobin (g/dl)	12.8 (12.6-13.0)	13.9 (13.7-14.1)	<0.0001
Hematocrit (%)	39.2 (38.7-39.7)	41.3 (40.9-41.8)	<0.0001
Platelets (10^3^/*μ*l)	309 (298-320)	237 (229-245)	<0.0001
Creatinine (*μ*M)	74 (72-76)	68 (66-69)	0.028
D-dimer (ng/ml)	477 (402-552)	225 (217-233)	<0.0001
Fibrinogen (g/l)	3.16 (3.10-3.25)	2.47 (2.24-3.10)	<0.0001
Plasminogen (%)	102.8 (101.0-104.6)	104.8 (102.9-106.6)	0.79
HRG (*μ*g/ml)	37.0 (36.2-37.9)	68.4 (66.5-70.2)	<0.0001
*Fibrin clot characteristics*			
*K*_s_ (10^−9^ cm^2^)	6.65 (6.54-6.76)	9.05 (8.84-9.26)	<0.0001
Lag phase (s)	39.0 (38.6-39.4)	42.5 (40-46)	<0.0001
ΔAbs (405 nm)	0.85 (0.84-0.86)	0.80 (0.79-0.81)	<0.0001
Clot lysis time (min)	99.0 (97.2-100.8)	75.5 (73.7-77.3)	<0.0001

Data are given as number (percentage), mean ± SD, or median (interquartile range). Abbreviations: WBC: white blood cells; HRG: histidine-rich glycoprotein; *K*_s_: fibrin clot permeability coefficient.

**Table 2:** Determinants of histidine-rich glycoprotein serum levels in lung cancer patients.

	Simple regression		Multiple regression		
	*β*	*p*	*β*	*p*	*R* ^2^
*Lung cancer patients*					
*K*_s_	0.09	0.27	-0.13	<0.001	0.19
CLT	-0.41	<0.001	-0.42		
Fibrinogen	-0.18	0.03	-0.15		
*Stage IIIAB/LD lung cancer patients*					
*K*_s_	-0.09	0.71	-0.09	0.014	0.17
CLT	-0.41	0.001	-0.44		
Fibrinogen	-0.09	0.48	-0.00		
*Stage IV/ED lung cancer patients*					
*K*_s_	0.28	0.008	0.07	<0.001	0.26
CLT	-0.5	<0.001	-0.46		
Fibrinogen	-0.18	0.09	-0.06		

*K*_s_: fibrin clot permeability coefficient; CLT: clot lysis time; LD: limited disease; ED: extensive disease.

## Data Availability

The data used to support the findings of this study are available from the corresponding author upon request.
